# Specialized minimal PDFs for optimized LHC calculations

**DOI:** 10.1140/epjc/s10052-016-4042-8

**Published:** 2016-04-15

**Authors:** Stefano Carrazza, Stefano Forte, Zahari Kassabov, Juan Rojo

**Affiliations:** 1 TIF Lab, Dipartimento di Fisica, Università di Milano, Via Celoria 16, Milan, 20133 Italy; 2 Sezione di Milano, INFN, Via Celoria 16, Milan, 20133 Italy; 3 Theory Department, CERN, Geneva, 1211 Switzerland; 4 Dipartimento di Fisica, Università di Torino, Via Pietro Giuria 1, Turin, 10125 Italy; 5 Sezione di Torino, INFN, Via Pietro Giuria 1, Turin, 10125 Italy; 6 Rudolf Peierls Centre for Theoretical Physics, University of Oxford, 1 Keble Road, OX1 3NP Oxford, UK

## Abstract

We present a methodology for the construction of parton distribution functions (PDFs) designed to provide an accurate representation of PDF uncertainties for specific processes or classes of processes with a minimal number of PDF error sets: specialized minimal PDF sets, or SM-PDFs. We construct these SM-PDFs in such a way that sets corresponding to different input processes can be combined without losing information, specifically as regards their correlations, and that they are robust upon smooth variations of the kinematic cuts. The proposed strategy never discards information, so that the SM-PDF sets can be enlarged by the addition of new processes, until the prior PDF set is eventually recovered for a large enough set of processes. We illustrate the method by producing SM-PDFs tailored to Higgs, top-quark pair, and electroweak gauge boson physics, and we determine that, when the PDF4LHC15 combined set is used as the prior, around 11, 4, and 11 Hessian eigenvectors, respectively, are enough to fully describe the corresponding processes.

## Introduction

Modern sets of parton distributions (PDFs) [1–6] provide a representation of their associated uncertainties based on either the Hessian [7] or the Monte Carlo (MC) [8] methods, supplementing their central PDF member with additional error members (eigenvectors or MC replicas). The number of PDF members required for an accurate representation of PDF uncertainty can be as large as several hundreds, especially when constructing PDF sets based on the combination of several underlying PDFs fitted to data: for example, the recent PDF4LHC 2015 sets [9] are based on a combined sample of 900 MC PDF replicas.

The usage of such large PDF samples can be computationally unwieldy, and this motivated the development of strategies for reducing the number of PDF members while minimizing accuracy loss. A number of such reduction strategies have been made available recently. Two of these methods provide a Hessian representation of the prior PDF set in terms of a smaller number of eigenvectors: META-PDFs [10] and MCH-PDFs [11]. A third method uses a compression algorithm to reduce the number of replicas of an underlying MC PDF prior: CMC-PDFs [12].

These three methods have been extensively benchmarked in the context of the 2015 PDF4LHC recommendations [9], where it was found that generally a set of about 100 PDFs is required in order to represent PDF uncertainties with percentage accuracy for all PDFs in the complete range of (*x*, *Q*) relevant for LHC phenomenology. However, it is well known [13] that, if one is interested only in the description of a specific set of cross sections, the number of PDF error members can be greatly reduced without significant accuracy loss.

In this work we propose a new strategy to achieve this goal. Our methodology, which we denote by Specialized Minimal PDFs (SM-PDFs), is based on the Singular Value Decomposition version of the mc2hessian algorithm, as presented in the appendix of Ref. [11]. Starting from either a Hessian or a Monte Carlo prior set and a list of collider processes, the SM-PDF algorithm leads to a set of eigenvectors optimized for the description of the input processes within some given tolerance.

In comparison to existing methods, such as data set diagonalization [13], our methodology has the advantage that no information is lost in the process of the construction of the specialized set. This is because the specialized set is constructed through a suitable linear transformation, whereby the starting space is separated into a subspace spanned by the optimized SM-PDF set, and its orthogonal subspace. This then implies that any given SM-PDF set can be iteratively expanded in order to maintain a given accuracy for an increasingly large set of processes, and also that SM-PDF sets optimized for different sets of processes can be combined into a single set, either a priori, at the level of PDFs, or a posteriori, at the level of cross sections. This, for example, enables the a posteriori combination of previous independent studies for a signal process and its corresponding backgrounds, with all correlations properly accounted for.

This paper is organized as follows: in Sect. 2 we describe our general strategy and methodology in detail. Then, in Sect. 3, we apply our method to the most important Higgs production channels (*ggh*, $ht\bar{t}$ and *hV*, VBF *h*) as well as for other standard candles at the LHC, i.e. $t\bar{t}$, *Z*, and *W* production. We compute one specific reduced sets for each of them, as well as a single set for all the processes combined. We validate the results by comparing the predictions of these reduced sets to the prior input set. We also show that our method provides an adequate generalization by showing that the predictions are stable when computing similar processes but with different kinematical cuts from those used as input. In Sect. 4 we show how experimental analyses done with different SM-PDFs can be combined together. In Sect. 5 we provide an overview of the deliverables of this work, in particular the code itself which allows one to easily generate reduced sets with personalized configuration and the LHAPDF6 [14] sets of SM-PDFs for the processes described in Sect. 5. Finally, Appendix A presents a graphical illustration of the regions in PDF space which give the dominant contribution to various physical processes, and Appendix B provides some basic instructions for the execution of the SM-PDF code.

## Methodology

The SM-PDF methodology is built upon the strategy based on Singular-Value Decomposition (SVD) followed by Principal Component Analysis (PCA) described in the appendix of Ref. [11], in which the MCH method was presented. This SVD+PCA strategy achieves the twofold goal of obtaining a multigaussian representation of a starting (prior) Monte Carlo PDF set, and of allowing for an optimization of this representation for a specific set of input cross sections, which uses the minimal number of eigenvectors required in order to reach a desired accuracy goal. We will now review the SVD+PCA method, and describe how it can be used for the construction of specialized minimal PDF sets, optimized for the description of a specific set of cross sections.

### The SVD+PCA method

The main problem we are addressing is the faithful representation of PDF uncertainties, which typically requires a large number of PDF error or Monte Carlo sets. Here we will assume the central value to be the same as in the prior PDF set, from which, if the prior is given as a Monte Carlo, it is typically determined as a mean (though different choices, such as the median, are possible and might be advisable in particular circumstances).

Hence, we are interested in the construction of a multigaussian representation in PDF space: the only information we need is then the corresponding covariance matrix. This is constructed starting with a matrix *X* which samples over a grid of points the difference between each PDF replica, $f_{\alpha }^{(k)}(x_{i},Q)$, and the central set, $f_{\alpha }^{(0)}(x_{i},Q)$, namely

1 $$\begin{aligned} X_{lk}(Q)\equiv f_{\alpha }^{(k)}(x_{i},Q)-f_{\alpha }^{(0)}(x_{i},Q), \end{aligned}$$

where $\alpha$ runs over the $N_f$ independent PDF flavors at the factorization scale $\mu _F=Q$, *i* runs over the $N_x$ points in the *x* grid where the PDFs are sampled, $l= N_{x}(\alpha -1)+i$ runs over all $N_{x}N_{f}$ grid points, and *k* runs over the $N_{\text {rep}}$ replicas. The sampling is chosen to be fine-grained enough that results will not depend on it.

The desired covariance matrix in PDF space is then constructed as

2 $$\begin{aligned} \text {cov}(Q) = \frac{1}{N_\mathrm{rep}-1}XX^t. \end{aligned}$$

The key idea which underlies the SVD method is to represent the $(N_{x}N_{f})\times (N_{x}N_{f})$ covariance matrix Eq. (2) over the $N_\mathrm{rep}$ dimensional linear space spanned by the replicas (assuming $N_\mathrm{rep}>N_{x}N_{f}$), by viewing its $N_{x}N_{f}$ eigenvectors as orthonormal basis vectors in this space, which can thus be represented as linear combinations of replicas. The subsequent PCA optimization then simply consists of picking the subspace spanned by the dominant eigenvectors, i.e., those with largest eigenvalues.

The first step is the SVD of the sampling matrix *X*, namely

3 $$\begin{aligned} X=USV^t, \end{aligned}$$

where *U* and $V^t$ are orthogonal matrices, with dimensions respectively $N_x N_f\times N_\mathrm{eig}^{(0)}$ and $N_\mathrm{rep} \times N_\mathrm{rep}$, *S* is a diagonal $N^{(0)}_\mathrm{eig}\times N_\mathrm{rep}$ positive semi-definite matrix, whose elements are the so-called singular values of *X*, and the initial number of singular values is given by $N^{(0)}_\mathrm{eig}=N_xN_f$. Note that, because *S* is diagonal, it can be equivalently viewed as a $N^{(0)}_\mathrm{eig}\times N^{(0)}_\mathrm{eig}$ matrix, since (with $N^{(0)}_\mathrm{eig}>N_\mathrm{rep}$) all its further entries vanish. This point of view was taken in the appendix of [11]. In this case, only the $N^{(0)}_\mathrm{eig} \times N_\mathrm{rep}$ submatrix which actually contributes to the SVD of the matrix *V* is included. However, for the procedure to be described below, it is more convenient to view *V* as $N_\mathrm{rep} \times N_\mathrm{rep}$ orthogonal matrix.

The matrix $Z=US$ then has the important property that

4 $$\begin{aligned} ZZ^t=X X^t, \end{aligned}$$

but also that it can be expressed as

5 $$\begin{aligned} Z=XV, \end{aligned}$$

and thus it provides the sought-for representation of the multigaussian covariance matrix in terms of the original PDF replicas: specifically, $V_{kj}$ is the expansion coefficient of the *j*th eigenvector over the *k*th replica. We assume henceforth that the singular values are ordered, so that the first diagonal entry of *S* correspond to the largest value, the second to the second-largest and so forth.

The PCA optimization then consists of only retaining the principal components, i.e. the largest singular values. In this case, *U* and *S* are replaced by their sub-matrices, denoted by *u* and *s*, respectively, with dimension $N_x N_f\times N_\mathrm{eig}$ and $N_\mathrm{eig}\times N_\mathrm{rep}$, with $N_\mathrm{eig} < N_\mathrm{eig}^{(0)}$ the number of eigenvectors which have been retained. Due to the ordering, these are the upper left sub-matrices. Because *s* has only $N_\mathrm{eig}$ non-vanishing diagonal entries, only the $N_\mathrm{rep}\times N_\mathrm{eig}$ submatrix of *V* contributes. We call this the principal submatrix *P* of *V*:

6 $$\begin{aligned} P_{kj}= V_{kj}, \quad k=1,\ldots ,N_\mathrm{rep},\quad j=1,\ldots ,N_\mathrm{eig}. \end{aligned}$$

The optimized representation of the original covariance matrix, Eq. (2), is then found by replacing *V* with its principal submatrix *P* in Eq. (5). This principal matrix *P* is thus the output of the SVD+PCA method: it contains the coefficients of the linear combination of the original replicas or error sets which correspond to the principal components, which can be used to compute PDF uncertainties using the Hessian method.

Indeed, given a certain observable $\sigma _i$ (which could be a cross section, the value of a structure function, a bin of a differential distribution, etc.) its PDF uncertainty can be computed in terms of the original Monte Carlo replicas by

7 $$\begin{aligned} s_{\sigma _i}= & {} \left( \frac{1}{N_\mathrm{rep}-1}\sum _{k=1}^{N_\mathrm{rep}}\left( \sigma _i^{(k)} -\sigma _i^{(0)}\right) ^2\right) ^\frac{1}{2}\nonumber \\= & {} \frac{1}{\sqrt{N_\mathrm{rep}-1}}\left\| d(\sigma _i)\right\| , \end{aligned}$$

where $\sigma _i^{(k)}$ is the prediction obtained using the *k*th Monte Carlo PDF replica, $\sigma _i^{(0)}$ is the central prediction, and in the last step we have defined the vector of differences

8 $$\begin{aligned} d_k(\sigma _i) \equiv \sigma _i^{(k)} - \sigma _i^{(0)},\quad k=1,\ldots ,N_\mathrm{rep}, \end{aligned}$$

with norm

9 $$\begin{aligned} \left\| d(\sigma _i)\right\| \equiv \left( \sum _{k=1}^{N_\mathrm{rep}}d_k^2(\sigma _i) \right) ^\frac{1}{2}. \end{aligned}$$

Assuming linear error propagation and using Eq. (5), the norm of the vector $\{ d_k(\sigma _i)\}$, Eq. (8), can be represented in the eigenvector basis:

10 $$\begin{aligned} \left\| d(\sigma _1)\right\| = \left\| {d^V}(\sigma _1)\right\| \end{aligned}$$

where the rotated vector

11 $$\begin{aligned} {d^V}_j(\sigma _i) = \sum _{k=1}^{N_\mathrm{rep}} d_k(\sigma _i) V_{kj}, \quad j=1,\ldots ,N^{(0)}_\mathrm{eig} \end{aligned}$$

has the same norm as the original one because of Eq. (4).

Replacing *V* by the principal matrix *P* in Eq. (11), i.e., letting *j* only run up to $N_\mathrm{eig}<N^{(0)}_\mathrm{eig}$ we get

12 $$\begin{aligned} \tilde{s}_{\sigma _i} = \frac{1}{\sqrt{N_\mathrm{rep}-1}} \left\| {d^P}(\sigma _i)\right\| , \end{aligned}$$

where now the vector is both rotated and projected

13 $$\begin{aligned} {d^P}_j(\sigma _i) = \sum _{k=1}^{N_\mathrm{rep}} d_k(\sigma _i) P_{kj}, \quad j=1,\dots ,N_\mathrm{eig}. \end{aligned}$$

The norm of $d^P$ is only approximately equal to that of the starting vector of differences *d*: $\left\| d^P(\sigma _1)\right\| \approx \left\| {d}(\sigma _1)\right\|$. However, it is easy to see that this provides the linear combination of replicas which minimizes the difference in absolute value between the prior and final covariance matrix for the given number of eigenvectors. As the difference decreases monotonically as $N_\mathrm{eig}$ increases, the value of $N_\mathrm{eig}$ can be tuned to any desired accuracy goal, with the exact equality Eq. (10) achieved when $N_\mathrm{eig}=N_\mathrm{eig}^{(0)}$. Note that, of course, the optimization step can be performed also starting with a symmetric Hessian, rather than Monte Carlo, prior. In such a case, the index *k* runs over Hessian eigenvectors, Eq. (2) is replaced by $\text {cov}(Q) = XX^t$, and the rest of the procedure is unchanged.

An interesting feature of this SVD+PCA method is that the matrix *V* (and thus also the principal matrix *P*) in Eq. (11) does not depend on the value of the PDF factorization scale *Q*: the scale dependence is thus entirely given by the DGLAP evolution equation satisfied by the original Monte Carlo replicas. The result of the SVD thus does not depend on the scale at which it is performed. Of course, the subsequent PCA projection may depend on scale if there are level crossings, but this is clearly a minor effect if a large enough number of principal components is retained. Because of this property, the SVD+PCA methodology can be used for the efficient construction [9] of a Hessian representation of combined PDF sets, even when the sets which enter the combination satisfy somewhat different evolution equations, e.g., because of different choices in parameters such as the heavy quark masses, or in the specific solution of the DGLAP equations.

### The SM-PDF method

In the SM-PDF method, this same SVD+PCA optimization is performed, but now with the goal of achieving a given accuracy goal not for the full prior PDF set in the complete range of *x* and $Q^2$, but rather for the aspects of it which are relevant for the determination of a given input set of cross sections, and in such a way that all the information which is not immediately used is stored and can be a posteriori recovered either in part or fully, e.g. if one wishes to add further observables to the input list.

This requires supplementing the SVD+PCA methodology of Ref. [11] with three additional features: a measure of the accuracy goal; a way of singling out the relevant part of the covariance matrix; and a way of keeping the information on the rest of the covariance matrix in such a way that, if needed, the full covariance matrix can be recovered at a later stage.

The main input to the algorithm is the set of $N_{\sigma }$ observables which we want to reproduce, $\{\sigma _i\}$, with $i=1,\ldots ,N_{\sigma }$. Theoretical predictions for the cross sections $\{\sigma _i\}$ are computed using a prior PDF set, which we assume for definiteness to be given as a Monte Carlo, though the method works with obvious modifications also if the starting PDFs are given in Hessian form. The goal of the SM-PDF methodology is to evaluate the PDF uncertainties $s_{\sigma _i}$, Eq. (7), in terms of a reduced number of Hessian eigenvectors,

14 $$\begin{aligned} \tilde{s}_{\sigma _i} = \left( \sum _{n=1}^{N_\mathrm{eig}}\left( \widetilde{\sigma }_i^{(n)} - \widetilde{\sigma }_i^{(0)}\right) ^2\right) ^\frac{1}{2}, \end{aligned}$$

with the number $N_\mathrm{eig}$ being as small as possible within a given accuracy. We thus define a measure $T_R$ of the accuracy goal (tolerance) by the condition

15 $$\begin{aligned} T<T_R;\qquad T \equiv \max _{i\in (1,N_{\sigma })} \Bigg | 1 - \frac{\tilde{s}_{\sigma _i}}{s_{\sigma _i}}\Bigg | \end{aligned}$$

in other words, $T_R$ is the maximum relative difference which is allowed between the original and reduced PDF uncertainties, $\tilde{s}_{\sigma _i}$ and $s_{\sigma _i}$, respectively, for all the observables $\{\sigma _i\}$.

In order to determine the part of the covariance matrix relevant for the description of the input observables $\{\sigma _i\}$, we define the correlation function

16 $$\begin{aligned}&\rho \left( x_i,Q, \alpha , \sigma _i\right) \nonumber \\&\quad \equiv \frac{N_\mathrm{rep}}{N_\mathrm{rep}-1} \left( \frac{\left\langle X(Q)_{lk} d_k(\sigma _i)\right\rangle _\mathrm{rep}- \left\langle X(Q_{\sigma _i})_{lk}\right\rangle _\mathrm{rep} \left\langle d_k(\sigma _i)\right\rangle _\mathrm{rep}}{s^\mathrm{PDF}_\alpha (x_i,Q) s_{\sigma _i} } \right) ,\nonumber \\ \end{aligned}$$

where the matrix of PDF differences *X*(*Q*) and the grid index $l=N_x(\alpha -1)+i$ have been defined in Eq. (1); $s_{\alpha }^\mathrm{PDF}(x_i,Q)$ is the standard deviation of the PDFs in the prior Monte Carlo representation, given by the usual expression,

17 $$\begin{aligned} s_{\alpha }^\mathrm{PDF}(x_i,Q) = \left( \frac{1}{N_\mathrm{rep}-1}\sum _{k=1}^{N_\mathrm{rep}} \left[ f_{\alpha }^{(k)}(x_i,Q)-\left\langle f_{\alpha }(x_i,Q)\right\rangle \right] \right) ^{\frac{1}{2}}, \end{aligned}$$

and $s_{\sigma _i}$, the standard deviation of the *i*th observable $\sigma _i$, is given by Eq. (7). The function Eq. (16) measures the correlation between the observables $\sigma _i$ and the *l*th PDF value (i.e. $f_{\alpha }(x_{i},Q)$, with $l=N_x(\alpha -1)+i$).

**Fig. 1:**
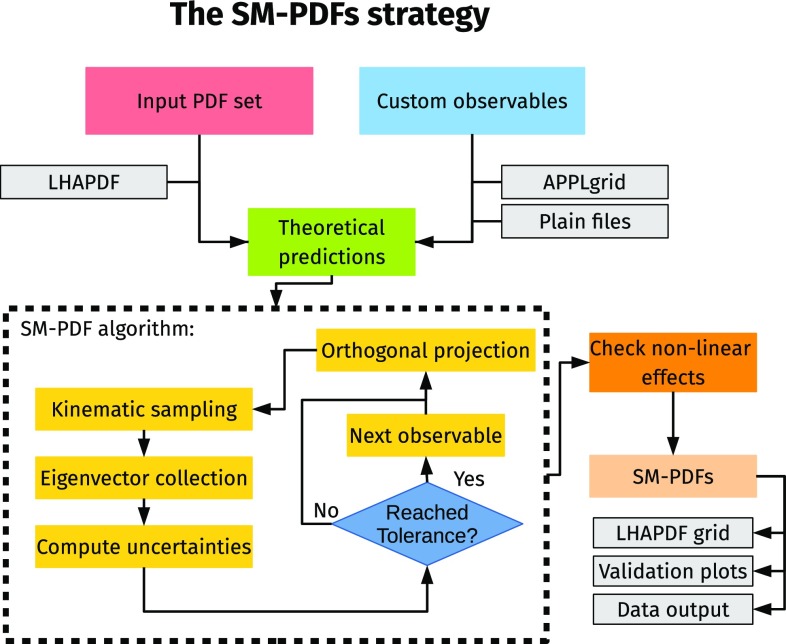
Schematic representation of the SM-PDF strategy

The basic idea of the SM-PDF construction is to apply the SVD to the subset of the covariance matrix which is most correlated to the specific observables that one wishes to reproduce, through a procedure such that information is never discarded, so observables can be added one at a time, or at a later stage. This goal is achieved through an iterative procedure schematically represented in Fig. 1, which we now describe in detail.

The iteration loop (contained in the dashed box in Fig. 1) is labeled by an iteration index *j*, such that at each iteration an extra eigenvector is added, thereby increasing the accuracy. If the accuracy goal is achieved for all observables after *j* iterations, then the final reduced Hessian set contains $N_\mathrm{eig}=j$ eigenvectors as error sets. These are obtained as a new principal matrix *P*, which provides the expansion coefficients of the eigenvectors over the replica basis: namely, $P_{kj}$ is the component of the *j*th eigenvector in terms of the *k*th replica. They thus replace the principal matrix of the previous PCA procedure as a final output of the procedure, and they can be used in exactly the same way.

To set off the iterative procedure, we select one of the observables we wish to reproduce from the list, $\sigma _1$, and compute the correlation coefficient $\rho \left( x_i,Q, \alpha , \sigma _1\right)$ for all grid points $(x_i,\alpha )$ and for a suitable choice of the scale *Q*. We then identify the subset $\Xi$ of grid points for which $\rho$ exceeds some threshold value:

18 $$\begin{aligned} \Xi =\left\{ (x_i,\alpha ): \rho \left( x_i,Q_{\sigma _1}, \alpha , \sigma _1\right) \ge t \rho _{\max } \right\} . \end{aligned}$$

The threshold value is expressed as a fraction $0<t<1$ times the maximum value $\rho _{\max }$ that the correlation coefficient takes over the whole grid, thereby making the criterion independent of the absolute scale of the correlation. The choice of the scale *Q* and the threshold parameter *t* should be taken as tunable settings of the procedure, and it will be discussed in Sect. 3 below. For the time being it suffices to say that *Q* should be of the order of the typical scale of the observable (for example, the average value of the factorization scale).

We then construct a reduced sampling matrix $X_{\Xi }$, defined as in Eq. (1), but now only including points in the $\{x_i, \alpha \}$ space which are in the subset $\Xi$. We perform the SVD of the reduced matrix

19 $$\begin{aligned} X_\Xi =USV^t, \end{aligned}$$

and we only keep the largest principal component, i.e. one single largest eigenvector, which is specified by the coefficients of its expansion over the replica basis, namely, assuming that the singular values are ordered, by the first row of the *V* matrix. We thus start filling our output principal matrix *P* by letting

20 $$\begin{aligned} P_{kj} = V_{k1}^{(j)}, \quad j=1, \quad k=1,\ldots ,N_\mathrm{rep}. \end{aligned}$$

Note that *j* on the left-hand side labels the eigenvector ($P_{kj}$ provides expansion coefficients for the *j*th eigenvector) and on the right-hand side it labels the iteration ($V_{k1}^{(j)}$ is the first row of the *V*-matrix at the *j*th iteration), which we can identify because, as mentioned, at each iteration we will add an eigenvector. The remaining eigenvectors of the principal matrix span the linear subspace orthogonal to *P*, and we assign them to a residual matrix *R*:

21 $$\begin{aligned} R_{km}^{(j)}= & {} V_{k(m+1)}^{(j)}\quad j=1, \nonumber \\ m= & {} 1,\ldots ,N_\mathrm{rep}-1, \quad k=1,\ldots ,N_\mathrm{rep}. \end{aligned}$$

At the first iteration, when there is only one eigenvector, the principal matrix *P* has just one row, and it coincides with the principal component of *V*. So far, the procedure is identical to that of the SVD+PCA method, and we can thus use again Eq. (12) to compute uncertainties on observables, check whether the condition Eq. (15) is met, and if it is not, add more eigenvectors. The procedure works in such a way that each time a new eigenvector is selected, exactly the same steps are repeated in the subspace orthogonal to that of the previously selected eigenvectors, thereby ensuring that information is never discarded. This is achieved by a projection method.

Specifically, we project the matrix *X* and the vector of observable differences $\{d_k(\sigma _i)\}$ on the orthogonal subspace of *P*, namely, the space orthogonal to that spanned by the eigenvectors which have already been selected (as many as the number of previous iterations). The projections are performed by, respectively, replacing $d(\sigma _i)$ and *X* by

22 $$\begin{aligned}&d^R(\sigma _i) =d(\sigma _i) R^{(j-1)},\end{aligned}$$

23 $$\begin{aligned}&X^R =X R^{(j-1)}, \end{aligned}$$

where the first iteration of the residual matrix $R^{(1)}$ has been defined in Eq. (21).

After the projection, we proceed as in the first iteration. We first determine again the subset $$\Xi $$, Eq. (18), of the projected sampling matrix $$ X^R$$, thereby obtaining a new sampling matrix $$X^R_\Xi $$: this is possible because everything is expressed as a linear combination of replicas anyway. Once the new matrix $$X^R_\Xi $$ has been constructed, the procedure is restarted from Eq. (19), leading to a new matrix $$V^R$$. The number of columns of the projected matrix $$X^R_\Xi $$ (and therefore of $$V^R$$) is $$N_\mathrm{rep}-(j-1)$$, which is the dimension of the subspace of the linear combinations not yet selected by the algorithm (that is, $$N_\mathrm{rep}-1$$ for $$j=2$$, and so on). We can now go back to Eq. (20) and proceed as in the previous case, but with the projected matrices: we add another row to the matrix of coefficients to the principal matrix by picking the largest eigenvector of the projected matrix, and determining again the orthogonal subspace.

At the *j*th iteration, this procedure gives

24 $$\begin{aligned} {P^R}^{(j)}_{k}&= {V^R}_{k1}^{(j)}, \quad k=1,\ldots ,N_\mathrm{rep}-(j-1),\end{aligned}$$

25 $$\begin{aligned} {R^R}_{km}^{(j)}&={V^R}_{k(m+1)}^{(j)}, \quad m=1,\ldots ,N_\mathrm{rep}-j,\nonumber \\&k=1,\ldots ,N_\mathrm{rep}-(j-1). \end{aligned}$$

which, respectively, generalize Eqs. (20) and (21) for $j\ge 1$. The projected row of coefficients $P^R$ Eq. (24) can be used to determine the corresponding unprojected row of coefficients of the principal matrix and of the residual matrix by using the projection *R* matrix in reverse, i.e., at the *j*th iteration

26 $$\begin{aligned} P^{(j)}_{kh}&=\sum _{k'} R^{(j-1)}_{k k'} {P^R}^{(j)}_{k'h} \, ,\end{aligned}$$

27 $$\begin{aligned} R^{(j)}_{kh}&=\sum _{k'} R^{(j-1)}_{kk'} {R^R}^{(j)}_{k'h}\, . \end{aligned}$$

We thus end up with a principal matrix which has been filled with a further eigenvector, and a new residual matrix and thus a new projection.

In summary, at each iteration we first project onto the residual subspace, Eq. (22), then pick the largest eigenvector in the subspace, Eq. (24), and then re-express the results in the starting space of replicas, Eq. (26), so that *P* is always the first row of *V* in each subspace, and Eqs. (13)–(12) remain valid as the *P* matrix is gradually filled. Determining the correlation and thus $\Xi$ after projection ensures that only the correlations with previously unselected linear combinations are kept. The fact that we are always working in the orthogonal subspace implies that the agreement for the observables $\sigma _i$, which had already been included, can only be improved and not deteriorated by subsequent iterations. It follows that we can always just check the tolerance condition on one observable at a time. The procedure is thus unchanged regardless of whether we are adding a new observable or not. In any case, the subset $\Xi$ for Eq. (18) is always determined by only one observable, namely, the one that failed to satisfy the tolerance condition at the previous iteration. The procedure is iterated until the condition is satisfied for all observables $\{\sigma _i\}$ in the input list. The number of iterations *j* until convergence defines the final number of eigenvectors $N_\mathrm{eig}$.

The output of the algorithm is the final $N_\mathrm{rep}\times N_\mathrm{eig}$ principal matrix *P*, which can be used to compute uncertainties on observables using Eqs. (12)–(13). However, for the final result we wish to obtain a set of Hessian eigenvectors. These can be obtained by performing the linear transformation given by *P* (a rotation and a projection) in the space of PDFs. The *X* matrix Eq. (1) then becomes

28 $$\begin{aligned} \widetilde{X} \equiv \sqrt{\frac{1}{N_\mathrm{rep}-1}}X P, \end{aligned}$$

so, substituting in Eq. (1), the final $N_\mathrm{eig}$ eigenvectors are found to be given by

29 $$\begin{aligned} \widetilde{f}_{\alpha }^{(k)}(x_i,Q)= f_{\alpha }^{(0)}(x_i,Q) + \widetilde{X}_{lk}(Q), \quad k=1,\ldots ,N_\mathrm{eig}. \end{aligned}$$

This is the same result as with the SVD+PCA algorithm of Sect. 2.1, but now generally with a smaller number of eigenvectors, namely, those which are necessary to describe the subset of the covariance matrix which is correlated to the input set of observables.

### SM-PDF usage and optimization

Upon delivery of the final PDF set, any observable is computed in terms of the resulting Hessian representation Eq. (29). As in the case of the original SVD+PCA methodology, the final result Eq. (29) determines the PDFs for all *x* and *Q*. Indeed, Eq. (29) determines the SM-PDF Hessian eigenvectors as linear combinations of replicas, and thus for all values of *x* and *Q* for which the original replicas were defined.

Note, however, that in the procedure of Sect. 2.2, in order to test for the tolerance criterion observables have been computed using Eqs. (12)–(13). This is equivalent to using the PDFs Eq. (28) by standard linear error propagation, but it differs from it by nonlinear terms, specifically for hadron collider processes in which observables are quadratic in the PDFs. Even though nonlinear corrections are expected to be small, in principle it could be that the tolerance criterion is no longer satisfied if Eq. (28) is used instead.

We explicitly check for this, and if it is the case for all observables $\sigma _i$ such that the recomputed tolerance criterion is not satisfied, we restart the iteration but now replacing the tolerance with a new value $T_{R,i}^\mathrm{(new)}$ given by

30 $$\begin{aligned} T_{R,i}^\mathrm{(new)} \equiv T_R - \left( T_i - T_i^\mathrm{(lin)} \right) , \end{aligned}$$

where $T_i^\mathrm{(lin)}$ is the value of the tolerance that is obtained within the linear approximation, by computing Eq. (15) with Eq. (12). Iterating until the criterion with the new tolerances Eq. (30) is met will be sufficient to ensure that the tolerance criterion is satisfied when using the new PDFs, provided the difference between the linear and exact estimate of $T_i$ is mostly due to the larger eigenvectors that were selected first and remains approximately constant upon addition of smaller eigenvectors in order to correct for this.

In practice, the difference between the linear estimation of the PDF uncertainty and the exact result is generally small, and does not a change the result for target tolerances $T_R$ of 5 % or bigger. This effect can be more important for observables affected by substantial PDF uncertainties, or for processes which depend on a large number of partonic channels (especially when new channels open up at NLO or NNLO). It is, however, not an issue for most practical applications.

Note that this final optimization step may become extremely time consuming if fast grid tools are not available. In view of this, it is possible to disable this check. However, fast interfaces can be obtained for any NLO QCD cross section with arbitrary final-state cuts using the aMCfast interface [15] to Madgraph5_aMC@NLO [16].

The SM-PDF construction can be generally performed at any perturbative order, and specifically starting with an NLO or an NNLO PDF set. The perturbative order enters both in the choice of starting PDF set, and in the computation of the list of observables $\{\sigma _i\}$, specifically used for the determination of the correlation function $\rho$ defined in Eq. (16). Because the NNLO-NLO *K* factors are usually moderate, for most applications it may be sufficient to compute $\rho$ using NLO theory even when using NNLO PDFs throughout. An obvious exception is the case in which the user is explicitly interested in studying the changes in PDFs when going from NLO to NNLO.

A final issue is whether results depend on the order in which the observables are included, and specifically on the choice of the observable $\sigma _1$ used to start the iteration. Indeed, the eigenvectors selected for a specific iteration depend on the subspace spanned by the previous eigenvectors, and consequently a different ordering will indeed change the particular linear combinations that are selected. However, this does not significantly affect the total number of eigenvectors needed, because the optimal subspace of linear combinations required to describe all observables with a given accuracy remains the same regardless of the order they are presented. We have verified that this is indeed the case, though we observed small fluctuations by one or two units in the final number of eigenvectors due to the discontinuous nature of the tolerance criteria, Eq. (15).

## Results and validation

We now present the validation of the SM-PDF algorithm described in the previous section. Using this methodology, we have constructed four specialized minimal PDF sets for different representative cases of direct phenomenological relevance at the LHC:

1. Higgs physics.
2. Top quark pair production physics.
3. Electroweak gauge boson production physics.
4. The combination of all processes included in (1), (2), and (3).

These examples have been chosen since, for each SM-PDF, there is a strong case for the use of optimized PDF sets with a greatly reduced number of eigenvectors. For instance, these SM-PDFs could be of interest for studies of the Higgs Cross-Section Working Group [17] (case 1), the LHC Top Working Group (case 2), and the LHC Electroweak Working Group (case 3), respectively. As an example, the SM-PDFs for *W*, *Z* production could be relevant for the determination of the *W* boson mass [18–20], which is a extremely CPU-time consuming task.

In this section, we will first define the PDF priors and LHC cross sections that have been used to construct the SM-PDF sets listed above, then validate the performance of the algorithm using a variety of figures of merit.

### Input PDFs and cross sections

In order to validate the SM-PDF methodology, we have used three different prior PDF sets, all of them in the Monte Carlo representation:

1. The NNPDF3.0 NLO set [6] with $N_\mathrm{rep}=1000$ replicas,
2. The MMHT14 NLO set [5] with $N_\mathrm{rep}=1000$ replicas, obtained from the native Hessian representation using the Watt-Thorne method [21], and
3. The PDF4LHC 2015 NLO prior set [9], with $N_\mathrm{rep}=900$ replicas, built from the combination of 300 replicas from each of the CT14, MMHT14 and NNPDF3.0 NLO sets. This set is denoted by MC900 in the following.

These three choices are representative enough for the validation of our methodology; they show that the procedure works regardless of the choice of input PDF set. As already mentioned in Sect. 2.3 the SM-PDF methodology can be applied equally to NLO or NNLO PDFs, and NLO PDFs are chosen here purely for the sake of illustration. Indeed, in Appendix B we provide an example in which NNLO PDFs are used.

**Table 1:** LHC processes and the corresponding differential distributions that have been used as input in the construction of the SM-PDFs dedicated to Higgs physics. In each case we also provide the APPLgrid grid name, the range spanned by each distribution, the number of bins $N_\mathrm{bins}$, and the kinematical cuts applied to the final-state particles. For associated production with vector bosons, *hW* and *hZ*, we impose basic acceptance cuts on the charged leptons from the weak boson decays. All processes have been computed for the LHC 13 TeV

Process	Input cross sections for SM-PDFs for Higgs physics				
	Distribution	Grid name	$N_{\mathrm{bins}}$	Range	Kin. cuts
$gg\rightarrow h$	incl xsec	ggh_13tev	1	–	–
	$d\sigma /dp_t^h$	ggh_pt_13tev	10	[0,200] GeV	–
	$d\sigma /dy^h$	ggh_y_13tev	10	[$-$2.5,2.5]	–
VBF *hjj*	incl xsec	vbfh_13tev	1	–	–
	$d\sigma /dp_t^h$	vbfh_pt_13tev	5	[0,200] GeV	–
	$d\sigma /dy^h$	vbfh_y_13tev	5	[$-$2.5,2.5]	–
*hW*	incl xsec	hw_13tev	1	–	$p_{T}(l)\ge 10$ GeV, $|\eta ^{l}|\le 2.5$
	$d\sigma /dp_t^h$	hw_pt_13tev	10	[0,200] GeV	$p_{T}(l)\ge 10$ GeV, $|\eta ^{l}|\le 2.5$
	$d\sigma /dy^h$	hw_y_13tev	10	[$-$2.5,2.5]	$p_{T}(l)\ge 10$ GeV, $|\eta ^{l}|\le 2.5$
*hZ*	incl xsec	hz_13tev	1	–	$p_{T}(l)\ge 10$ GeV, $|\eta ^{l}|\le 2.5$
	$d\sigma /dp_t^h$	hz_pt_13tev	10	[0,200] GeV	$p_{T}(l)\ge 10$ GeV, $|\eta ^{l}|\le 2.5$
	$d\sigma /dy^h$	hz_y_13tev	10	[$-$2.5,2.5]	$p_{T}(l)\ge 10$ GeV, $|\eta ^{l}|\le 2.5$
$ht\bar{t}$	incl xsec	httbar_13tev	1	–	–
	$d\sigma /dp_t^h$	httbar_pt_13tev	10	[0,200] GeV	–
	$d\sigma /dy^h$	httbar_y_13tev	10	[$-$2.5,2.5]	–

In order to compute the theoretical predictions for all input PDF sets and as many cross sections as possible, we have generated a large number of dedicated APPLgrid grids [22] using the aMCfast [15] interface to MadGraph 5_aMC@NLO [16]. Cross sections and differential distributions have been computed for the LHC Run II kinematics, with a center-of-mass energy of $\sqrt{s}=13$ TeV. In particular we have generated fast NLO grids for the following processes:

- *Higgs production* Total cross sections and rapidity and $p_T$ differential distributions for gluon fusion, vector-boson fusion, associated production with *W* and *Z* bosons and associated production with top-quark pairs. No Higgs decays are included, since we are only interested in the production dynamics.
- *Top quark pair production* Total cross section, $p_t$, and rapidity distributions of the top and the anti-top quarks, and invariant mass $m_{t\bar{t}}$, $p_t$, and rapidity distributions of the $t\bar{t}$ system.
- *Electroweak gauge boson production* For *Z* production: total cross section, $p_T$, and rapidity distributions of the two charged leptons and of the *Z* boson, and $p_T$ and invariant mass distribution of the dilepton pair. For *W* production: total cross section, $p_T$, and rapidity distributions of the charged lepton and of the *W* boson, missing $E_T$ and transverse mass $m_T$ distribution. For the *W* and *Z* processes, we apply kinematical cuts to the charged leptons from the weak boson decay to reflect the typical acceptance constraints of the LHC experiments.

A more detailed description of these processes, including binning and the kinematical cuts applied, is provided in Tables 1, 2, and 3. We also indicate the names of the (publicly available) APPLgrid grids generated for the present validation study. Producing fast NLO grids for additional processes, or with a different binning or set of analysis cuts, is straightforward using the aMC@NLO/aMCfast framework. We adopt the default choice of renormalization and factorization scales in aMC@NLO, namely $\mu _F=\mu _R=H_T/2$, with

31 $$\begin{aligned} H_T\equiv \sum _i \sqrt{p_{T,i}^2+m_i^2}, \end{aligned}$$

the scalar sum of the transverse masses of all final-state particles at the matrix-element level.

**Table 2:** Same as Table 1 for the SM-PDFs dedicated to top-quark pair production physics

Process	Input cross sections for SM-PDFs for $t\bar{t}$ physics				
	Distribution	Grid name	$N_{\mathrm{bins}}$	Range	Kin. cuts
$t\bar{t}$	incl xsec	ttbar_13tev	1	–	–
	$d\sigma /dp_t^{\bar{t}}$	ttbar_tbarpt_13tev	10	[40,400] GeV	–
	$d\sigma /dy^{\bar{t}}$	ttbar_tbary_13tev	10	[$-$2.5,2.5]	–
	$d\sigma /dp_t^{t}$	ttbar_tpt_13tev	10	[40,400] GeV	–
	$d\sigma /dy^{t}$	ttbar_ty_13tev	10	[$-$2.5,2.5]	–
	$d\sigma /dm^{t\bar{t}}$	ttbar_ttbarinvmass_13tev	10	[300,1000]	–
	$d\sigma /dp_t^{t\bar{t}}$	ttbar_ttbarpt_13tev	10	[20,200]	–
	$d\sigma /dy^{t\bar{t}}$	ttbar_ttbary_13tev	12	[$-$3,3]	–

**Table 3:** Same as Table 1 for the SM-PDFs dedicated to electroweak gauge boson production physics. The kinematical cuts are applied to the charged leptons from the weak boson decays

Process	Input cross sections for SM-PDFs for electroweak boson production physics				
	Distribution	Grid name	$N_{\mathrm{bins}}$	Range	Kin. cuts
*Z*	incl xsec	z_13tev	1	–	$p_{T}(l)\ge 10$ GeV, $|\eta ^{l}|\le 2.5$
	$d\sigma /dp_t^{l^-}$	z_lmpt_13tev	10	[0,200] GeV	$p_{T}(l)\ge 10$ GeV, $|\eta ^{l}|\le 2.5$
	$d\sigma /dy^{l^-}$	z_lmy_13tev	10	[$-$2.5,2.5]	$p_{T}(l)\ge 10$ GeV, $|\eta ^{l}|\le 2.5$
	$d\sigma /dp_t^{l^+}$	z_lppt_13tev	10	[0,200] GeV	$p_{T}(l)\ge 10$ GeV, $|\eta ^{l}|\le 2.5$
	$d\sigma /dy^{l^-}$	z_lpy_13tev	10	[$-$2.5,2.5]	$p_{T}(l)\ge 10$ GeV, $|\eta ^{l}|\le 2.5$
	$d\sigma /dp_t^{z}$	z_zpt_13tev	10	[0,200] GeV	$p_{T}(l)\ge 10$ GeV, $|\eta ^{l}|\le 2.5$
	$d\sigma /dy^{z}$	z_zy_13tev	5	[$-$4,4]	$p_{T}(l)\ge 10$ GeV, $|\eta ^{l}|\le 2.5$
	$d\sigma /dm^{ll}$	z_lplminvmass_13tev	10	[50,130] GeV	$p_{T}(l)\ge 10$ GeV, $|\eta ^{l}|\le 2.5$
	$d\sigma /dp_t^{ll}$	z_lplmpt_13tev	10	[0,200] GeV	$p_{T}(l)\ge 10$ GeV, $|\eta ^{l}|\le 2.5$
*W*	incl xsec	w_13tev	1	–	$p_{T}(l)\ge 10$ GeV, $|\eta ^{l}|\le 2.5$
	$d\sigma /d\phi$	w_cphi_13tev	10	[$-$1,1]	$p_{T}(l)\ge 10$ GeV, $|\eta ^{l}|\le 2.5$
	$d\sigma /dE_t^\mathrm{miss}$	w_etmiss_13tev	10	[0,200] GeV	$p_{T}(l)\ge 10$ GeV, $|\eta ^{l}|\le 2.5$
	$d\sigma /dp_t^{l}$	w_lpt_13tev	10	[0,200] GeV	$p_{T}(l)\ge 10$ GeV, $|\eta ^{l}|\le 2.5$
	$d\sigma /dy^{l}$	w_ly_13tev	10	[$-$2.5,2.5]	$p_{T}(l)\ge 10$ GeV, $|\eta ^{l}|\le 2.5$
	$d\sigma /dm_{t}$	w_mt_13tev	10	[0,200] GeV	$p_{T}(l)\ge 10$ GeV, $|\eta ^{l}|\le 2.5$
	$d\sigma /dp_t^{w}$	w_wpt_13tev	10	[0,200] GeV	$p_{T}(l)\ge 10$ GeV, $|\eta ^{l}|\le 2.5$
	$d\sigma /dy^{w}$	w_wy_13tev	10	[$-$4,4]	$p_{T}(l)\ge 10$ GeV, $|\eta ^{l}|\le 2.5$

Clearly, some of these cross sections contain overlapping information, so our list is partially redundant. For instance, if differential distributions are reproduced, this will also be the case for total inclusive cross sections. Similarly, the rapidity distributions of the *W* and *Z* bosons are closely related to the rapidity distributions of the leptons from their decay, so including both distributions will lead to a certain degree of redundancy.

This redundancy can be used to provide a non-trivial check of our methodology. For instance, we have verified that by beginning with the total cross sections, only the most extreme bins of the differential distributions, which contribute less to the cross section, might require extra eigenvectors in order to be reproduced to the desired tolerance. Conversely, if we begin the algorithm using differential distributions as input, no additional eigenvectors are required to describe the corresponding total cross sections.

### Choice of settings

The SM-PDF method is fully determined by the choice of kinematic region $\Xi$, Eq. (18), which in turn is fully specified by the correlation function and tolerance $T_R$. The only tunable parameters are thus the scale *Q* used for the evaluation of correlations in Eq. (16) and the threshold value *t*. As the choice of the scale *Q*, we adopt the mean value of the factorization scale $\mu _F$ at which the PDFs are evaluated by the corresponding APPLgrid grids, that is, the event-by-event weighted average of the value of $\mu _F$ used in the calculation of each specific cross section or differential distribution.

The only remaining free parameter is then the threshold *t*, which specifies according to Eq. (18) which points are included in the reduced matrix $X|_{\Xi }$: low values of *t* lead to the inclusion of a wider region in phase space, and conversely. Clearly, if $\Xi$ is too wide, the reduction will not be very effective and the ensuing number of eigenvectors will be large. On the other hand, if the region $\Xi$ is too small, the number of eigenvectors will be small, but it might be lead to a result which is unstable upon small changes of the input observables.

**Fig. 2:**
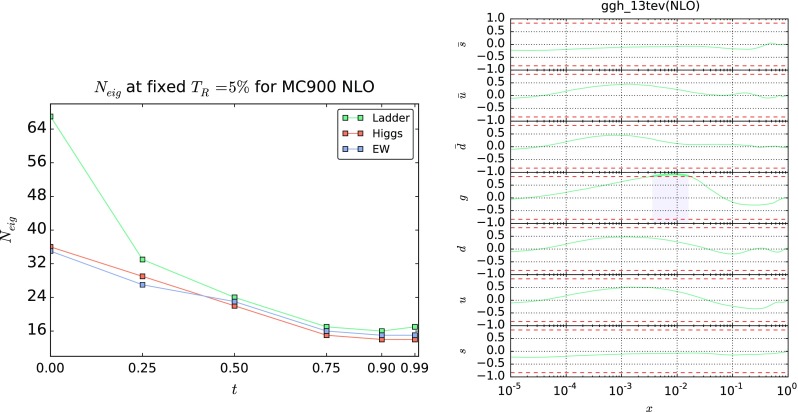
*Left* final number of eigenvectors $N_\mathrm{eig}$ obtained applying the SM-PDF algorithm to the MC900 NLO PDF set with 900 Monte Carlo replicas, as a function of the threshold parameter *t* Eq. (18) for fixed tolerance $T_R=5~\%$. We show the results for three choices of input cross sections: Higgs (Table 1), electroweak gauge boson production (Table 3), and “ladder” (all processes in Tables 1, 2, and 3). *Right* correlation Eq. (16) between all the PDFs and the total cross section for Higgs production in gluon fusion, as a function of *x* (*solid blue lines*). The value $\rho = 0.9\rho _\mathrm{max}$ is shown as a *dashed line* and the region in which the correlation exceeds the threshold is shown as a *shaded band*

In order to determine a suitable value of *t*, we use the full set of cross sections listed in Tables 1, 2, and 3. We will henceforth refer to this specific set of observables (and the associate SM-PDF set) as the “ladder”. In Fig. 2 (left) we plot the number of eigenvectors $N_\mathrm{eig}$, which we obtained as a function of the parameter *t* when the SM-PDF methodology is applied to the MC900 prior set, for a fixed tolerance $T_R=5~\%$. We show the results for the Higgs, EW and the “ladder” set of input processes.

As expected, $N_\mathrm{eig}$ decreases as the value of *t* is raised, since in this case fewer points in the $(\alpha ,x)$ grid are selected. While the specific position of the minimum of the $N_\mathrm{eig}(t)$ curve depends on the input set of cross sections, we see from Fig. 2 that the curve reaches its minimum around $t\sim 0.9$ for all processes. Note that, as discussed at the end of Sect. 2.3, the value of $N_\mathrm{eig}(t)$ can fluctuate, typically by one or two units, depending on the specific ordering of the input processes. We therefore choose $t=0.9$: this means that we adopt the smallest value of *t* (i.e. the widest kinematic region) compatible with having the smallest possible number of eigenvectors.

In Fig. 2 (right) we show the value of the correlation coefficient Eq. (16) between the MC900 prior set and the inclusive cross section for Higgs production in gluon fusion, as a function of *x* and for the seven independent PDF flavors, evaluated at the average scale *Q* of the grids. The value of the correlation $\rho = t\rho _\mathrm{max}$ corresponding to $t=0.9$ is shown as a dashed red line in the plots; the points for which the correlation coefficient (blue curve) is larger in modulus than the threshold are shown as a shaded region.

We observe that, for this specific cross section, the algorithm in the first iteration will include in the region $\Xi$ for Eq. (18) only the gluon PDF for $x\simeq 10^{-2}$, which corresponds to the region that dominates the total cross section for Higgs production in gluon fusion. In Appendix A we provide additional correlation plots, similar to Fig. 2 (right) but for other Higgs production channels, as well as the correlation plots for subsequent iterations, $j\ge 2$, of the algorithm, illustrating how the selected regions in the $\left( x,\alpha \right)$ grid vary along the iteration.

### Results and validation

We now present the results of applying the SM-PDF procedure to the PDF sets and cross sections described in Sect. 3.1. In Table 4 we show the results for the number of eigenvectors $N_\mathrm{eig}$ obtained, for each input PDF set, using the three different groups of LHC processes that we consider: Higgs, $t\bar{t}$, and *W* / *Z* production. In addition, for the Higgs production processes, we have also studied the results of applying our methodology to each of the Higgs production channels individually, as summarized in Table 5. The algorithm has been applied for two different values of the tolerance $T_R$, namely 5 and 10 %. We also indicate in the bottom row the results for the “ladder” SM-PDF (i.e. including all the above processes.)

**Table 4:** Number of eigenvectors $N_\mathrm{eig}$ obtained by applying the SM-PDF procedure, starting from each of the three input prior PDF sets, to the three families of processes summarized in Tables 1, 2, and 3: Higgs production, $t\bar{t}$ production, and *W* / *Z* production physics. The final row is based on the inclusion of all the three families of processes, in the same order as they are listed. Results are shown for two different values of the tolerance threshold $T_R$, 5 and 10 %, respectively

Process	$N_\mathrm{eig}$					
	MC900		NNPDF3.0		MMHT14	
	$T_R=5~\%$	$T_R=10~\%$	$T_R=5~\%$	$T_R=10~\%$	$T_R=5~\%$	$T_R=10~\%$
*h*	15	11	13	8	8	7
$t\bar{t}$	4	4	5	4	3	3
*W*, *Z*	14	11	13	8	10	9
ladder	17	14	18	11	10	10

**Table 5:** Same as Table 4, now for the case where the separate Higgs production channels as used as input to the SM-PDF algorithm

Process	$N_\mathrm{eig}$					
	MC900		NNPDF3.0		MMHT14	
	$T_R=5~\%$	$T_R=10~\%$	$T_R=5~\%$	$T_R=10~\%$	$T_R=5~\%$	$T_R=10~\%$
$gg\rightarrow h$	4	5	4	4	3	3
VBF *hjj*	7	5	10	5	4	3
*hW*	6	5	6	4	6	3
*hZ*	11	7	6	4	8	5
$ht\bar{t}$	3	2	4	4	3	2
Total *h*	15	11	13	8	8	7

Several comments on Table 4 are in order.

- Results are reasonably stable upon a change of tolerance, with differences smaller with the MMHT14 prior, which has smaller underlying number of parameters than NNPDF3.0.
- The most dramatic reduction in number of eigenvectors is seen for the production of top pairs, or Higgs in gluon fusion, where only $N_\mathrm{eig} \simeq 4$ eigenvectors are needed. This can be understood as a consequence of the fact that in both cases the dominant contribution to the cross section arises from the gluon distribution in a narrow region of *x*.
- Total cross sections and differential distributions for all the Higgs production modes can be reproduced, in the case of the MC900 prior, with 11 to 15 eigenvectors (depending on the choice of tolerance $T_R$).
- The number of eigenvectors required is largest for the Higgs and the *W* / *Z* family of processes, as one would expect given that in both cases several PDFs in a wide kinematic range are required.
- All the processes that we are including can be described with a SM-PDF set, the “ladder”, which includes about the same number of eigenvectors as needed for the Higgs or for the Drell–Yan and *W* / *Z* family of processes. This “ladder” SM-PDF, with only $N_\mathrm{eig}\simeq$ 15 eigenvectors, can be used reliably for a large number of LHC cross sections, including those not included in its construction.

**Fig. 3:**
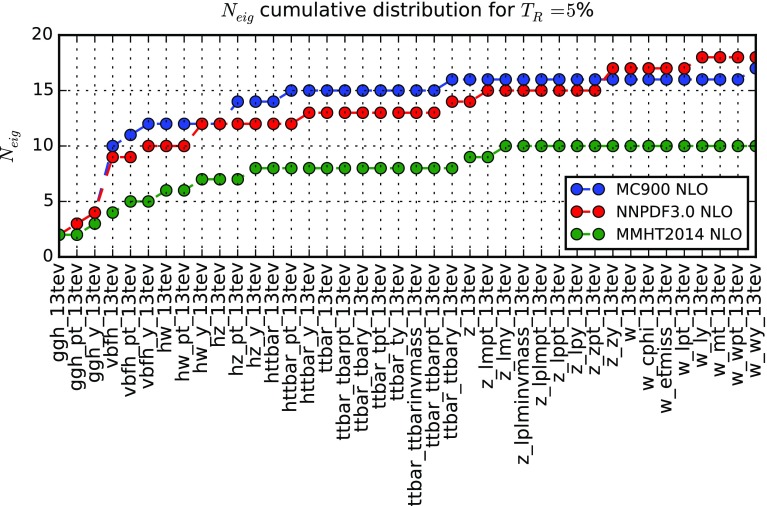
Total number of eigenvectors $N_\mathrm{eig}$ required by the SM-PDF algorithm to describe a sequentially increasing number of input cross sections and distributions, for a tolerance $T_R=5~\%$. Results are presented for the three prior PDF sets, namely MC900, NNPDF3.0, and MMHT14

Next, in Fig. 3 we show the total number of eigenvectors $N_\mathrm{eig}$, which are required, for a tolerance of $T_R=5~\%$, as more and more processes are sequentially included, until the complete list of processes in Tables 1, 2, and 3 has been exhausted. This plot demonstrates the robustness and flexibility of the SM-PDF algorithm, in that it shows how more processes can be added without information loss to a reduced PDF set, thereby allowing for a study of the information brought in by each process. In Fig. 3 results are presented for the three input PDF sets, MC900, NNPDF3.0 and MMHT14. As already seen in Table 4, a smaller number of eigenvectors is required in order to describe the MMHT14 set, which has a smaller underlying number of parameters than the NNPDF3.0 set; the combined MC900 set requires roughly the same number of eigenvectors as NNPDF3.0, which is contained in it. Inspection of Fig. 3 indicates which processes bring in new information in comparison to those already included. For instance, the fact that the number of eigenvectors is unchanged when adding all the observables related to top-quark pair production shows that SM-PDFs based on Higgs processes also describe top production.

**Fig. 4:**
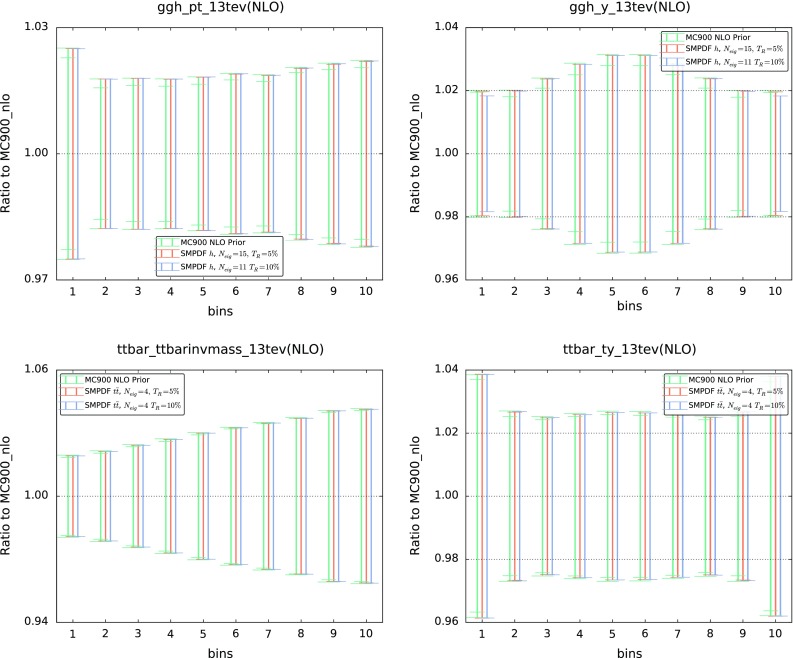
*Upper plots* comparisons of the predictions for the $p_t$ (*left*) and rapidity (*right*) differential distributions in Higgs production in gluon fusion between the prior MC900 and the corresponding Higgs SM-PDFs for two different values of the tolerance $T_R$, 5 and 10 %. Results are shown normalized to the central value of MC900. *Lower plots* same comparison, now for the $t\bar{t}$ SM-PDFs, showing the invariant mass of the $t\bar{t}$ pair $m_{t\bar{t}}$ (*left*) and the top-quark rapidity $y^t$ (*right*). See Tables 1 and 2 for the details of the binning and the kinematical cuts in each case

**Fig. 5:**
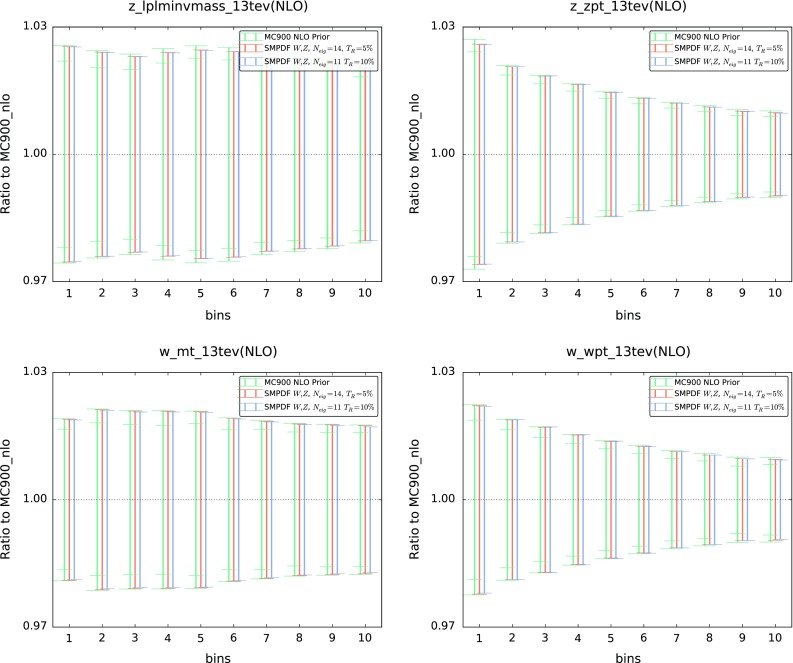
Same as Fig. 4 for representative differential distributions in *W* and *Z* production, comparing the MC900 prior with the *W*, *Z* SM-PDFs

**Fig. 6:**
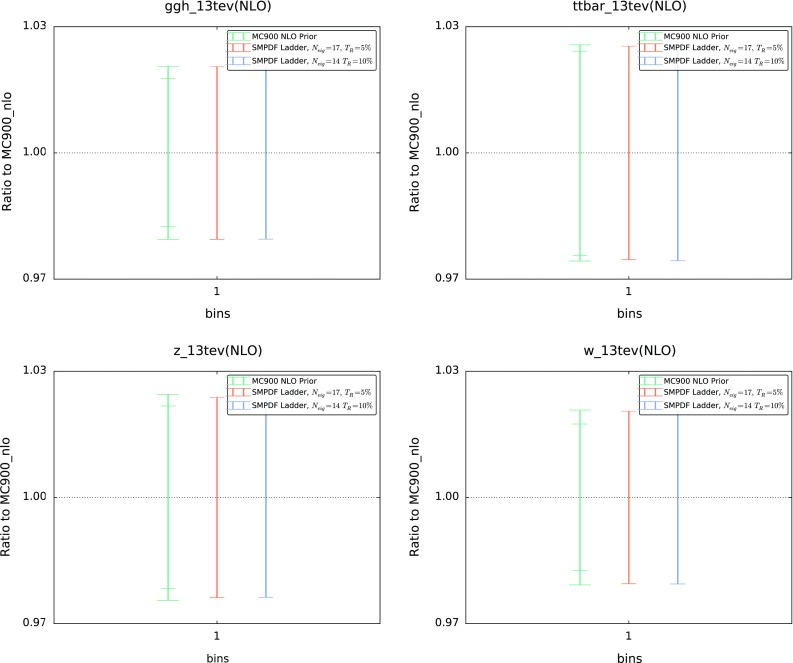
Same as Fig. 4 for the “ladder” SM-PDF, now comparing with the total *ggH*, $t\bar{t}$, *Z*, and *W* inclusive cross sections

In Figs. 4, 5, 6 we compare various cross sections and differential distributions computed with the MC900 prior PDF set and with the corresponding SM-PDFs for some of the cases discussed above, normalized to the central value of the prior. In the upper plots of Fig. 4, we show the Higgs $p_T$ and *y* distributions in gluon fusion production, comparing with the Higgs SM-PDF. In the lower plots of Fig. 4, we show the top-quark pair invariant mass $m_{t\bar{t}}$ and top rapidity $y^t$ distributions, comparing with the $t\bar{t}$ SM-PDF. In Fig. 5 we compare various differential distributions in weak gauge boson production with the *W*, *Z* SM-PDFs, and finally in Fig. 6 we compare the “ladder” SM-PDFs with various total inclusive cross sections.

In these comparisons, results are shown for two values of the tolerance, $T_R=5~\%$ and $T_R=10~\%$. PDF uncertainties are shown as one-sigma confidence intervals; for the MC900 prior, the central 68 % confidence intervals are also shown (inner ticks). In all cases we observe excellent agreement between the prior and the corresponding SM-PDF sets, which provides a further validation of the reliability of the method.

We have also verified that SM-PDFs reproduce well PDF correlations, even though the tolerance criterion Eq. (15) is only imposed on diagonal PDF uncertainties. The PDF-induced correlation between two cross sections computed using a Monte Carlo PDF set is given by

32 $$\begin{aligned} \rho (\sigma _i,\sigma _j) = \frac{\left<\sigma _1^{(k)}\sigma _2^{(k)}\right>_\mathrm{rep} - \left<\sigma _1^{(k)}\right>_\mathrm{rep}\left<\sigma _2^{(k)}\right>_\mathrm{rep}}{s_{\sigma _1}s_{\sigma _2}}, \end{aligned}$$

while for a Hessian set it is

33 $$\begin{aligned} \rho (\sigma _i,\sigma _j) = \frac{ \sum _{k=1}^{N_\mathrm{eig}}\left( \widetilde{\sigma }^{(k)}_i- \sigma ^{(0)}_i \right) \left( \widetilde{\sigma }^{(k)}_j- \sigma ^{(0)}_j \right) }{\widetilde{s}_{\sigma _1}\widetilde{s}_{\sigma _2}}. \end{aligned}$$

**Fig. 7:**
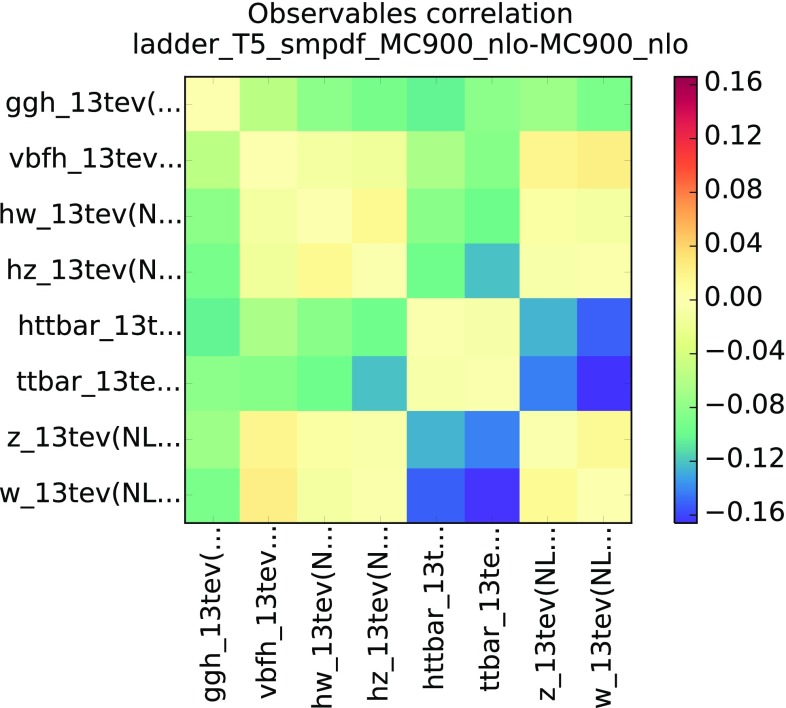
Differences in the correlation coefficients between the MC900 prior and the “ladder” SM-PDFs with $T_R=5~\%$, computed for all the inclusive cross sections that enter the construction of the latter

In Fig. 7 we show the difference between the correlations determined using the MC900 prior (from Eq. (32)) and the “ladder” SM-PDF set (from Eq. (33)), with $T_R=5~\%$, for all the total inclusive cross sections used as input to the “ladder” SM-PDF set. We find that the deviation in correlation is at the few percent level or better for most cases, and anyway never worse than 20 %.

**Fig. 8:**
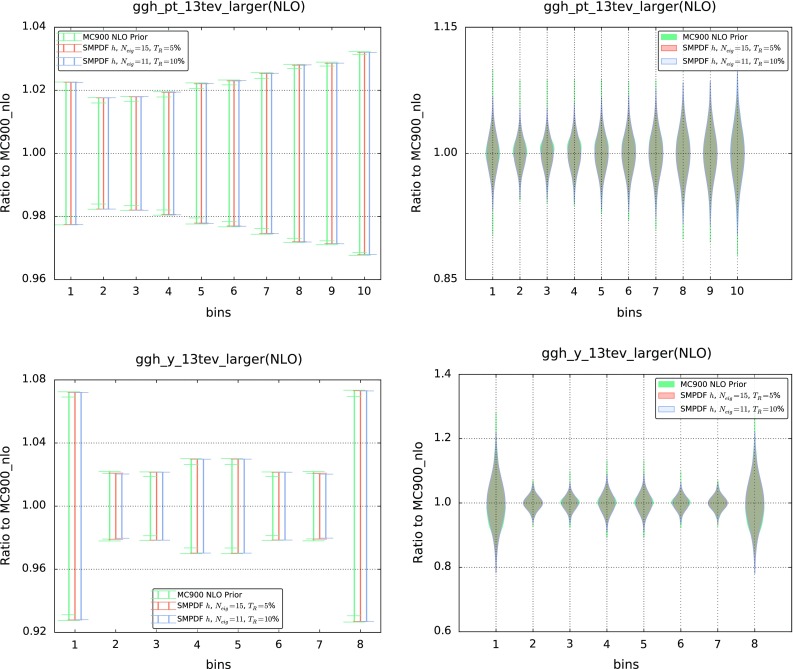
The $p_t$ and rapidity distributions for Higgs production in gluon fusion, computed with the MC900 prior and with the Higgs SM-PDFs, for two values of the tolerance $T_R$, this time in a kinematic range that doubles that of the input processes in Table 1 (see text). In the *left plot* we show the standard deviation in each bin, while in the *right plot* we show the full probability distributions per bin, reconstructed using the Kernel Density Estimate (KDE) method

An additional validation test can be performed by comparing the predictions for a given SM-PDF outside the kinematic range of the input processes. To illustrate this point, in Fig. 8 we compare the $p_t$ and rapidity distributions in Higgs production via gluon fusion using the Higgs SM-PDF (which uses as input the processes in Table 1) but now with an extended kinematical range: the rapidity distribution now includes $y \in \left[ -5,5 \right]$, rather than the range $y \in \left[ -2.5,2.5 \right]$ used as input, and the $p_t$ distribution covers now $p_t \in [0,400]$ GeV as compared to the original input $p_t \in [0,200]$ GeV. In both cases, we show both the standard deviation (left) and the full probability distribution obtained with the prior and the two compressed sets with $T_R=5~\%$ and $T_R=10~\%$; the smoothened probability distributions are obtained using the using the Kernel Density Estimation (KDE) method discussed in Ref. [12]. The good agreement seen in all cases demonstrates the robustness of the SM-PDF method: namely, SM-PDF sets are stable upon variations of kinematic cuts and binning of the input cross sections.

**Fig. 9:**
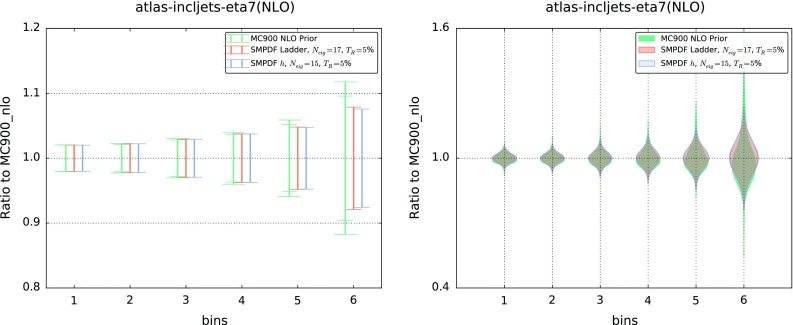
Same as Fig. 8, but now for the ATLAS inclusive jet $p_T$ distribution in the forward region, and using the “ladder” SM-PDF set

While the SM-PDFs are stable upon extrapolation, they will not provide accurate predictions when used for processes dominated by PDFs in an altogether different kinematic range. To illustrate this point, in Fig. 9 we show predictions for inclusive jet distributions obtained using the Higgs and ladder SM-PDF sets, compared to the result obtained using the MC900 prior. Specifically, we show the $p_t^\mathrm{jet}$ distributions in the most forward rapidity bin ($3.6 \le |y_\mathrm{jet}|<4.4$) of the ATLAS 2010 inclusive jet measurement [23]; bins are ordered in increasing $p_T$. Clearly, the agreement deteriorates at large $p_T$, where results depend on the large-*x* quarks and gluon, which are weakly correlated to the processes included in the construction of both the Higgs and the “ladder” SM-PDF sets. This also suggests that good agreement, with a marginally larger number of eigenvectors, could be likely obtained by just widening the range of some of the inputs to the “ladder”, such as, for instance, including the Higgs transverse-momentum distribution up higher values of $p_t$. In fact, we have explicitly checked [24] that the “ladder” PDF set provides comparable accuracy to the PDF4LHC15 30 eigenvector set when used for the determination of all the hadronic observables included in the NNPDF3.0 PDF determination [6], despite having almost half the number of eigenvectors.

## A posteriori combination of SM-PDFs

So far, we considered the construction of a PDF set tailored to a given list of input cross sections. However, one may also encounter the situation in which two SM-PDF sets constructed using different processes as input are already available, and wishes to use them simultaneously, without having to produce a new dedicated SM-PDF set using as input the two processes at the same time. A typical application is a computation in which one of these processes is the signal and the other to a background. The SM-PDF methodology also allows one to deal with this situation: we first discuss how this is done, and then we present an example of an application.

### General method

In Sect. 2 we have shown how, starting from a Monte Carlo PDF prior, $X_{lk}$, Eq. (1), we can construct a specialized minimal Hessian representation, $\widetilde{X}_{lk}$, Eq. (28), in terms of a reasonably small number of eigenvectors. The result of the SM-PDF algorithm can be expressed as a regular Hessian PDF set, with the error parameters given by Eq. (29). Alternatively, one can directly use the final matrix of Hessian coefficients *P* to express the cross sections computed with each of the replicas of the prior set, Eq. (8), as linear combinations of cross sections computed with the final eigenvector sets, Eq. (13). The two results are equivalent by linear error propagation.

However, we can also read Eq. (13) in reverse: if we define

34 $$\begin{aligned}&d_k^\mathrm{MC}(\sigma _i) = \sqrt{N_\mathrm{rep}-1}\,\, \sum _{j=1}^{N_\mathrm{eig}}P_{kj}{d^P}_j(\sigma _i), \nonumber \\&k=1,\ldots ,N_\mathrm{rep}, \quad i=1,\ldots ,N_{\sigma }, \end{aligned}$$

we can view the set of $N_\mathrm{rep}$ differences $d_k^\mathrm{MC}(\sigma _i)$ (for each of the $N_{\sigma }$ observables $\sigma _i$) as a Monte Carlo set of cross sections, containing the same information as the reduced SM-PDF set. In other words, the $N_\mathrm{rep}$ values

35 $$\begin{aligned}&\sigma ^{(k)}_i =\sqrt{N_\mathrm{rep}-1}\,\,\sum _{j=1}^{N_\mathrm{eig}}P_{kj}{d^P}_j(\sigma _{i})+\sigma _{i}^{(0)}, \nonumber \\&\quad k=1,\ldots ,N_\mathrm{rep}, \quad i=1,\ldots ,N_{\sigma }, \end{aligned}$$

of the observable $\sigma _i$ can be viewed as “pseudo-Monte Carlo” replicas, to be used to compute uncertainties and correlations using the standard Monte Carlo procedure.

If two sets of SM-PDFs corresponding to different processes are available, we can then combine the information contained in them by first turning the predictions obtained from them into replicas using Eq. (35), and then viewing the set of Monte Carlo replica predictions obtained in each case as our best approximation to the Monte Carlo set of predictions for that process obtained with the original PDF replica set. These sets of prediction replicas can then be used in order to compute any quantity which depends on both processes using the standard Monte Carlo methodology, by just making sure that each process is computed using its corresponding replicas.

### Validation

We illustrate and validate the methodology presented in Sect. 4.1 with an example. We use as input prior the NNPDF3.0 NLO set with $N_\mathrm{rep}=1000$ replicas and then generate two SM-PDFs for a fixed choice of the tolerance $T_R=5~\%$. The first SM-PDF takes as input the $t\bar{t}$ processes from Table 2, while the second is constructed from the *W*, *Z* processes of Table 3.

We now use these two SM-PDF sets to calculate the PDF uncertainties on the $t\bar{t}$ and the *W* total inclusive cross sections. This can be done both with the original representation, Eq. (7), or with the new SM-PDF Hessian representation. As shown in Table 4, we find $N_\mathrm{eig}=5$ for the $t\bar{t}$ SM-PDF and $N_\mathrm{eig}=13$ for the *W*, *Z* SM-PDF. We obtain the following results for the total cross sections: for the $t\bar{t}$ cross section with $t\bar{t}$ SM-PDFs

36 $$\begin{aligned} \sigma _{t\bar{t}\ (\mathrm{prior})} = 671.12\pm 12.0\ \mathrm{pb}, \end{aligned}$$

37 $$\begin{aligned} \sigma _{t\bar{t}\ (\mathrm{smpdf-tt})} = 671.12\pm 11.9\ \mathrm{pb}, \end{aligned}$$

and for the *W* cross section with *W*, *Z* SM-PDF

38 $$\begin{aligned} \sigma _{W\ (\mathrm prior)} = 23867\pm 419\ \mathrm{pb}, \end{aligned}$$

39 $$\begin{aligned} \sigma _{W\ (\mathrm smpdf-wz)} = 23867\pm 417\ \mathrm{pb}. \end{aligned}$$

Now suppose that we want to compute a quantity which depends both on the $t\bar{t}$ and the *W* cross sections, such as the ratio between the two, $\sigma _{t\bar{t}}/\sigma _W$. In the computation of the PDF uncertainty on this ratio, it is essential to properly account for the cross-correlations between the two processes. This can be achieved by recasting the results of the two different SM-PDFs into corresponding Monte Carlo sets of predictions through Eq. (35).

Namely, the PDF uncertainty on the cross-section ratio is given by

40 $$\begin{aligned} s_{\frac{\sigma _{t\bar{t}}}{\sigma _{W}}}= \frac{1}{N_\mathrm{rep}-1}\left( \sum _{k=1}^{N_\mathrm{rep}} \left( \frac{\sigma ^{(k)}_{t\bar{t}}}{\sigma ^{(k)}_{W}}- \left\langle \frac{\sigma ^{(k)}_{t\bar{t}}}{\sigma ^{(k)}_{W}} \right\rangle _\mathrm{rep} \right) ^{2}\right) ^{\frac{1}{2}}, \end{aligned}$$

where $\sigma ^{(k)}_{t\bar{t}}$ and $\sigma ^{(k)}_{W}$ have been obtained using Eq. (35) with the *P* matrix that corresponds, respectively, to the $t\bar{t}$ and *W*, *Z* SM-PDF sets.

Using Eq. (40) we get

41 $$\begin{aligned} s_{\frac{\sigma _{t\bar{t}}}{\sigma _{W}}}= 6.66497\times 10^{-4}, \end{aligned}$$

to be compared to the result obtained from the NNPDF3.0 prior, using the $N_\mathrm{rep}=1000$ original replicas,

42 $$\begin{aligned} s_{\frac{\sigma _{t\bar{t}}}{\sigma _{W}}(\mathrm{prior})}= 6.66503\times 10^{-4}, \end{aligned}$$

which is identical for all practical purposes.

It is important to realize that while Eq. (42) requires the calculation of $2N_\mathrm{rep}=2000$ cross sections, Eq. (41) only requires the knowledge of the $N_\mathrm{eig}$ cross section differences $\widetilde{d}_j(\sigma _i)$ for the two observables, which is equal to the sum of the number of eigenvectors in the two sets which are being combined; in our case, $N_\mathrm{eig}^{WZ}+N_\mathrm{eig}^{t\bar{t}}=18$, with great computational advantage.

As a further cross-check, we have recomputed the same cross section ratio by using the methodology of Sect. 2, namely, by constructing a dedicated SM-PDF set using as input the two families of processes, $t\bar{t}$ and *W*, *Z*, simultaneously.

This new SM-PDF has now 17 eigenvectors for the case of a tolerance $T_R=5~\%$ and leads to

43 $$\begin{aligned} s_{\frac{\sigma _{t\bar{t}}}{\sigma _{W}}(\mathrm{combined})}= 6.655\times 10^{-4}. \end{aligned}$$

This shows that the advantage of constructing a dedicated set in comparison to combining the pre-existing sets is marginal, as the accuracy is the same, and the total number of eigenvectors $N_\mathrm{eig}$ has only decreased by one unit.

## Delivery

Building upon our previous MC2H methodology for the construction of reduced Hessian representations of PDF uncertainties [11], we have presented an algorithm for the construction of a minimal Hessian representation of any given prior PDF set, specialized to reproduce a number of input cross sections. We have shown that the algorithm can be used to construct specialized minimal PDF sets which reproduce with percent accuracy the central values and PDF uncertainties for all input observables in terms of a substantially smaller number of eigenvectors as compared to the prior PDF set. A remarkable advantage of the SM-PDF methodology is that the complete information contained in the original prior set is kept at all stages of the procedure. As a consequence, it is possible to add new processes to any given SM-PDF set with no information loss. Also, it is possible to combine a posteriori SM-PDF sets corresponding to different processes without any new computation.

The SM-PDF code is publicly available from the repository

https://github.com/scarrazza/smpdf/

The code is written in Python using the numerical implementations provided by the NumPy package. Customized interfaces to APPLgrid and LHAPDF6 are also included. The package also includes the APPLgrid grids for all the processes listed in Tables 1, 2, and 3, and additional processes can be easily generated upon request.

The input of the SM-PDF code is the prior PDF set and the list of cross sections $\{ \sigma _i\}$ to be reproduced. The code settings can be modified by the user by means of a steering card. The cross sections can be provided either by indicating the name of the APPLgrid or by means of a text file (for predictions computed with external codes). An example steering card for the code is presented in Appendix B.

The output of the code is then the corresponding SM-PDF set, directly in the LHAPDF6 format, as well as the corresponding direct and inverse Hessian parameter matrices, *P* and $P^t$, respectively as a CSV file. These rotation matrices allow one to easily transform the computed cross sections back and forth from any SM-PDF representation to the prior representation, as well as transforming between different SM-PDF representations, as explained in Sect. 4.

Together with this, a number of additional validation features are included in the SM-PDF package. In particular, comparisons at the level of the input cross sections as those presented in Figs. 2, 4, and 7 can be generated automatically by filling the appropriate options in the YAML configuration file, without the need of writing additional code. The user is encouraged to refer to the documentation for a more extensive description of the different features available. In addition, a web interface to similar to that of APFEL Web on-line PDF plotter [25, 26] is currently under consideration.

Finally, the SM-PDFs constructed in Sect. 3 are also available from the same webpage in the LHAPDF6 format. Users can produce the SM-PDFs that more suitable for specific applications by generating the suitable cross section theory calculations and then running the SM-PDF code. However, users are encouraged to contact the authors for support if assistance is needed. Additional SM-PDFs can be added to this webpage upon request.

## Figures and Tables

**Fig. 10:**
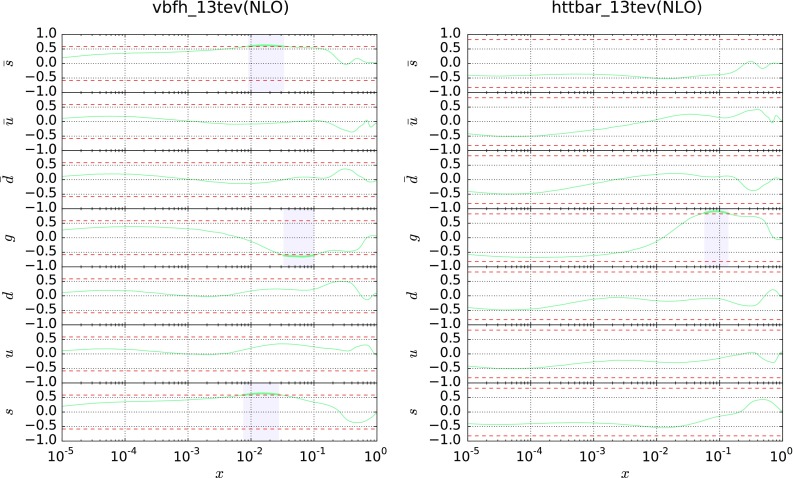
Correlation Eq. (16) between the PDFs and the cross section for Higgs production in vector-boson fusion (*left*) and associated with a $t\bar{t}$ pair (*right*), as a function of *x*, computed using the MC900 NLO PDF set. The threshold value $\rho = 0.9\rho _\mathrm{max}$ is shown as a *dashed line*, and the region in which the correlation coefficient exceeds the threshold, $\rho \ge 0.9\rho _\mathrm{max}$, is shown as a *shaded band*

**Fig. 11:**
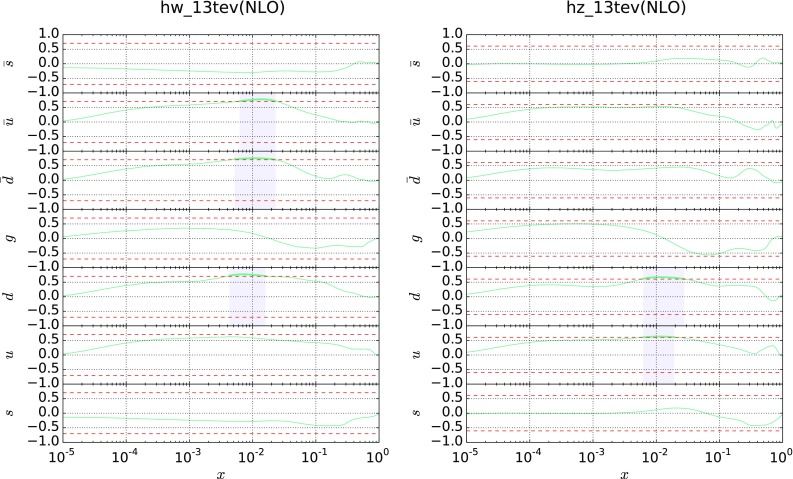
Same as Fig. 10 for associated production of Higgs bosons with *W* (*left*) and *Z* bosons (*right*)

**Fig. 12:**
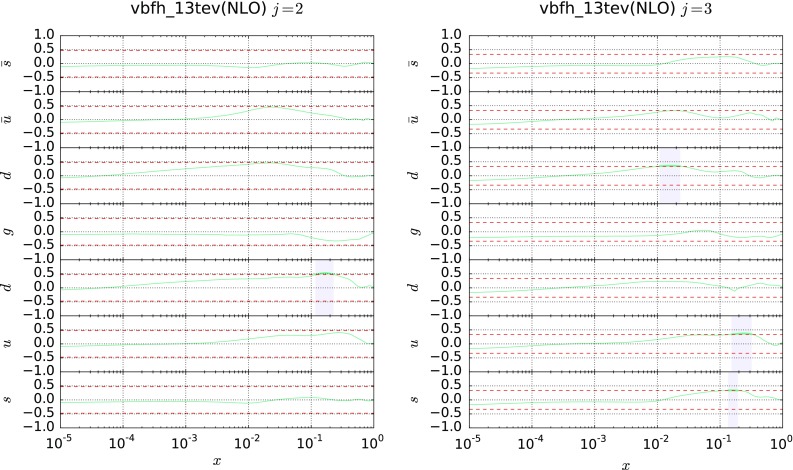
Same as the *left plot* of Fig. 10, but now at the second (*left*) and third (*right*) iteration of the SM-PDF algorithm

**Fig. 13:**
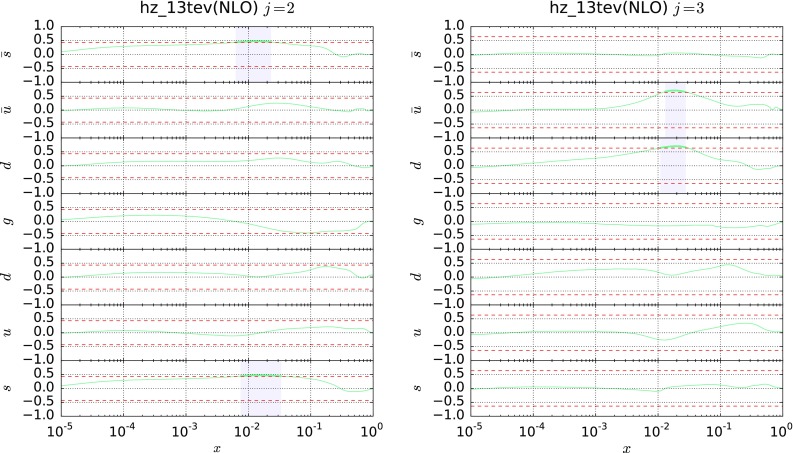
Same as Fig. 12, but now, in this case, the results for the first iteration of the algorithm were shown in the right plot of Fig. 11

## Appendix A: PDF correlations

In this appendix we illustrate graphically the selection of the region $\Xi$ Eq. (18) by the SM-PDF algorithm.

In Fig. 10 we plot as a function of *x* the value of the correlation Eq. (16) between PDFs and the total cross section for Higgs production in vector-boson fusion (VBF) and in association with a $t\bar{t}$ pair, determined using MC900 NLO PDFs. The $\Xi$ region is that in which the correlation exceeds the value $\rho = 0.9\rho _\mathrm{max}$, shown as a dashed line in the plots, and it is highlighted with a gray band.

The corresponding comparison for Higgs production in gluon fusion was shown in Fig. 2. We see that $\Xi$ includes the gluon around $x\simeq \left( 0.05,0.1\right)$ and the strangeness $s,\bar{s}$ around $x\simeq 10^{-2}$, while for $ht\bar{t}$ production it includes the gluon for $x \simeq 0.1$.

The corresponding comparisons for Higgs production in association with *W* and *Z* bosons is shown in Fig. 11. In this case, for *hW* the $\Xi$ region includes the $\bar{u}$, $\bar{d}$ and *d* quark PDFs for $x\simeq 10^{-2}$, and for *hZ* production the same region, but for the *u* and *d* quark PDFs.

The regions shown in Figs. 10 and 11 are selected at the first iteration of the SM-PDF algorithm. These are therefore the regions which are needed in order to determine the most important eigenvector. At the subsequent iteration, further regions are selected in the orthogonal subspace. The regions selected at the second and third iterations for Higgs production in VBF and *hZ* production are, respectively, shown in Figs. 12 (to be compared to the first iteration, shown in left plot of Fig. 11) and 13 (to be compared to the first iteration, shown in left plot of Fig. 11).

For VBF in the second iteration $\Xi$ contains the *d* PDF at $x\simeq 0.2$ and the third the *d* PDF at $x\simeq 0.02$, and the up and strange PDFs at $x\simeq 0.2$. For *hZ*, it contains the strange PDFs around $x\simeq 10^{-2}$ at the second iteration, and at the third iteration the $\bar{u}$ and $\bar{d}$ PDFs for $x \simeq \left( 0.01,0.05\right)$. In each case, there is no overlap between regions selected in subsequent iterations, as it must be because of the projection. The hierarchy in selection shows which regions and PDFs are increasingly less important in determining the given cross section.

## Appendix B: Basic usage of the SM-PDF code

While we refer the user to the documentation bundled in with the SM-PDF code, which will be updated over time, here we provide an annotated example of a basic YAML configuration file that can be used to define the inputs to the code. In particular, the following example of the steering card is the one used to generate the Higgs SM-PDF set, constructed using all the processes in Table 1 as input. In addition, the main executable also produces a number of validation plots such as those presented in Sect. 3.

Following installation, the SM-PDF code can be executed using the following command:

$$\begin{aligned} \mathtt {smpdf}\, \mathtt {higgs.yaml --use-db} \end{aligned}$$

where the steering card should contain the following information:

No additional settings need to be modified. By default, the code will also output the generated SM-PDF set directly in the LHAPDF6 format.
